# Supplementary figures and images for: TNF-α antagonists differentially induce TGF-β1-dependent resuscitation of dormant-like Mycobacterium tuberculosis
Source: PLoS Pathog. 2020 Feb 18;16(2):e1008312. doi: 10.1371/journal.ppat.1008312 (PMC7048311; doi:10.1371/journal.ppat.1008312)

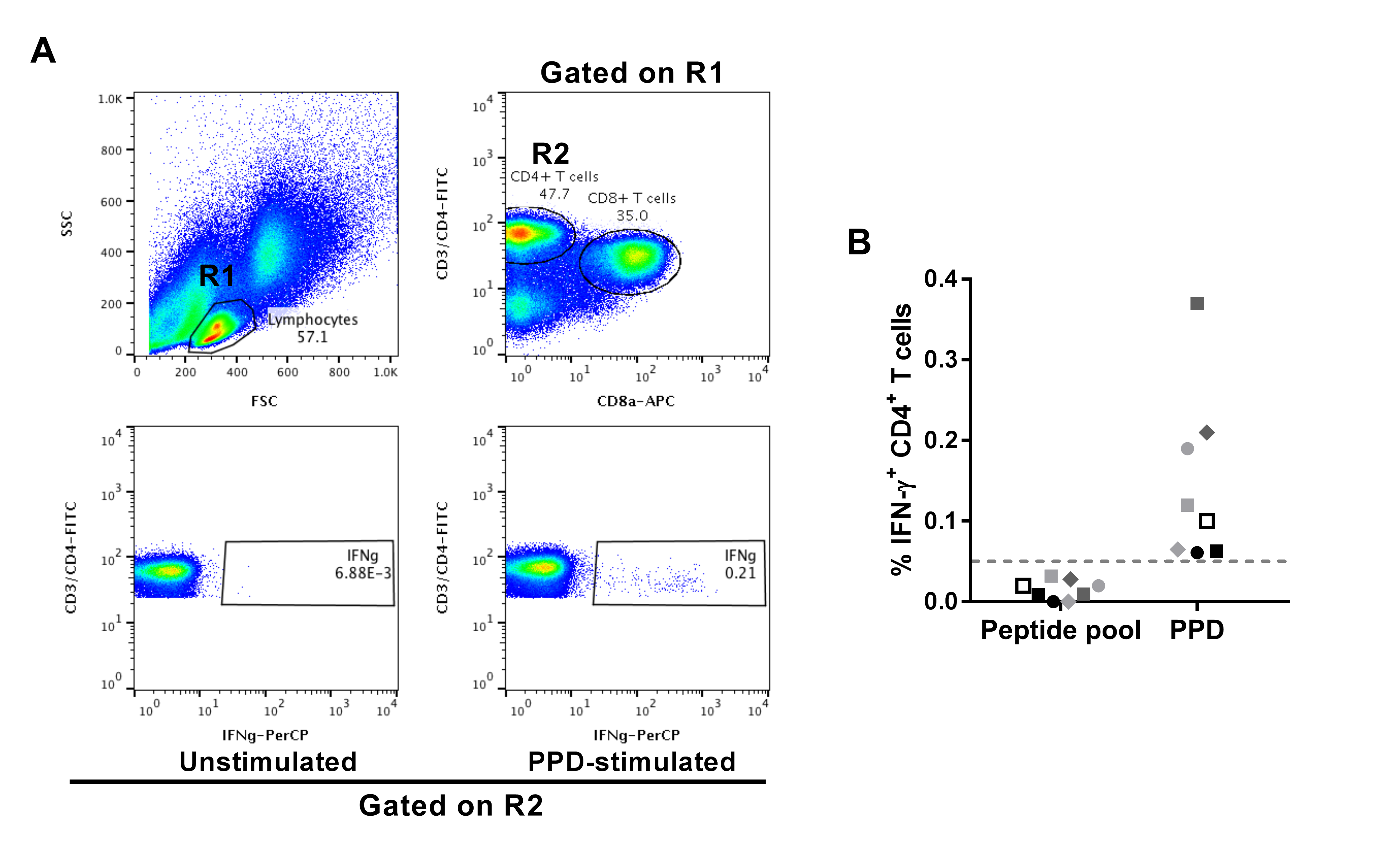

Supplement: S1 Fig — PBMCs were stimulated overnight with Mtb protein purified derivative (PPD) or a synthetic peptide pool from ESAT-6, CFP-10 and TB7.7 Mtb proteins and analyzed by flow cytometry. (A) Representative dotplots of the gating strategy. (B) Background-subtracted frequencies of IFN-γ-producing CD4+ T cells for each donor selected for the study. The response was considered positive when more than 0.05% of cytokine-producing cells were detected within the CD4+ T cell parent population and this frequency was at least twice higher than the background level detected in the absence of stimuli. (TIF) [file ppat.1008312.s001.tif]

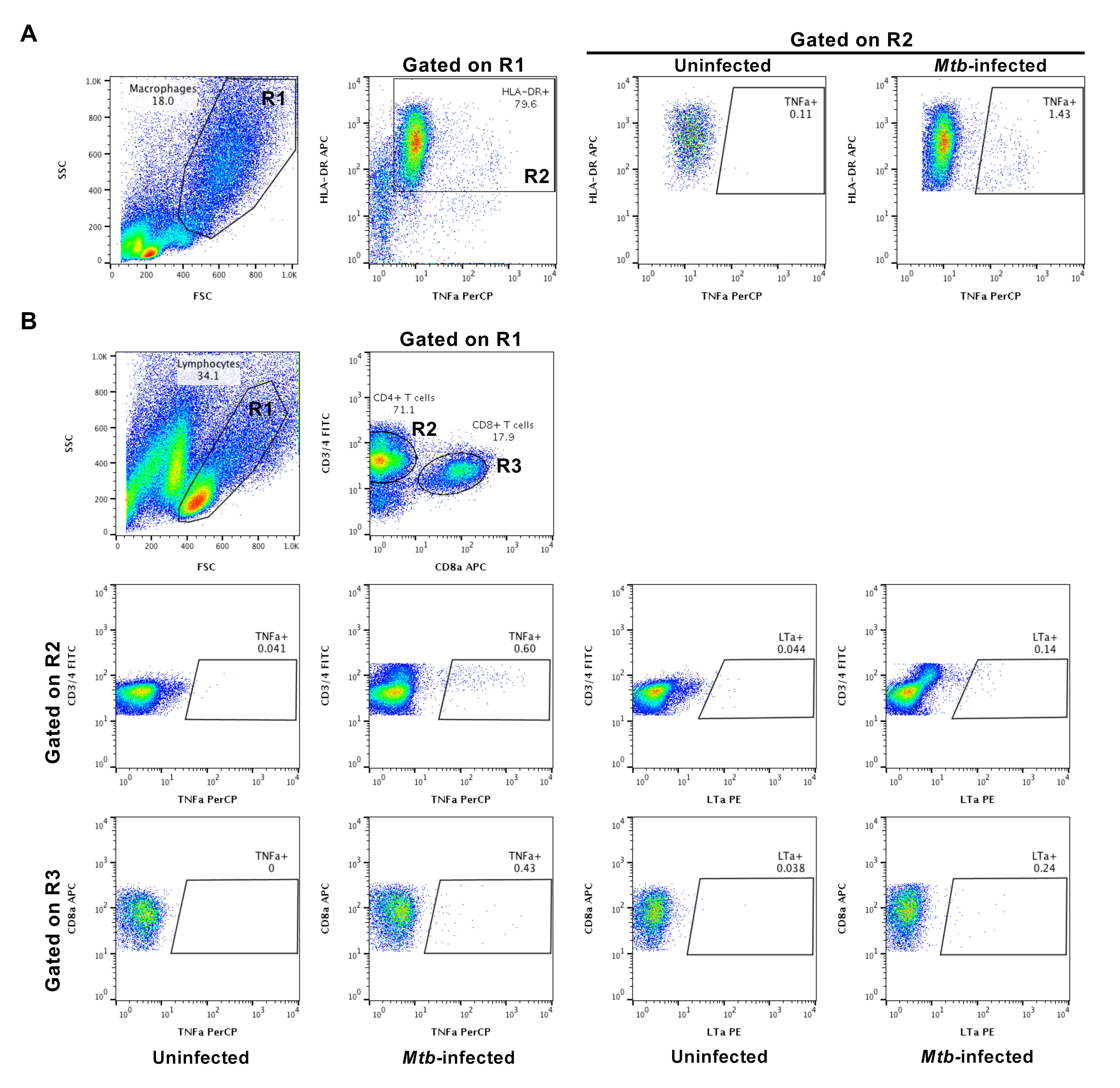

Supplement: S2 Fig — Representative dotplots showing the gating strategy used to focus on HLA-DR+ macrophage (A) or CD4+ and CD8+ T cell populations (B). (TIF) [file ppat.1008312.s002.tif]

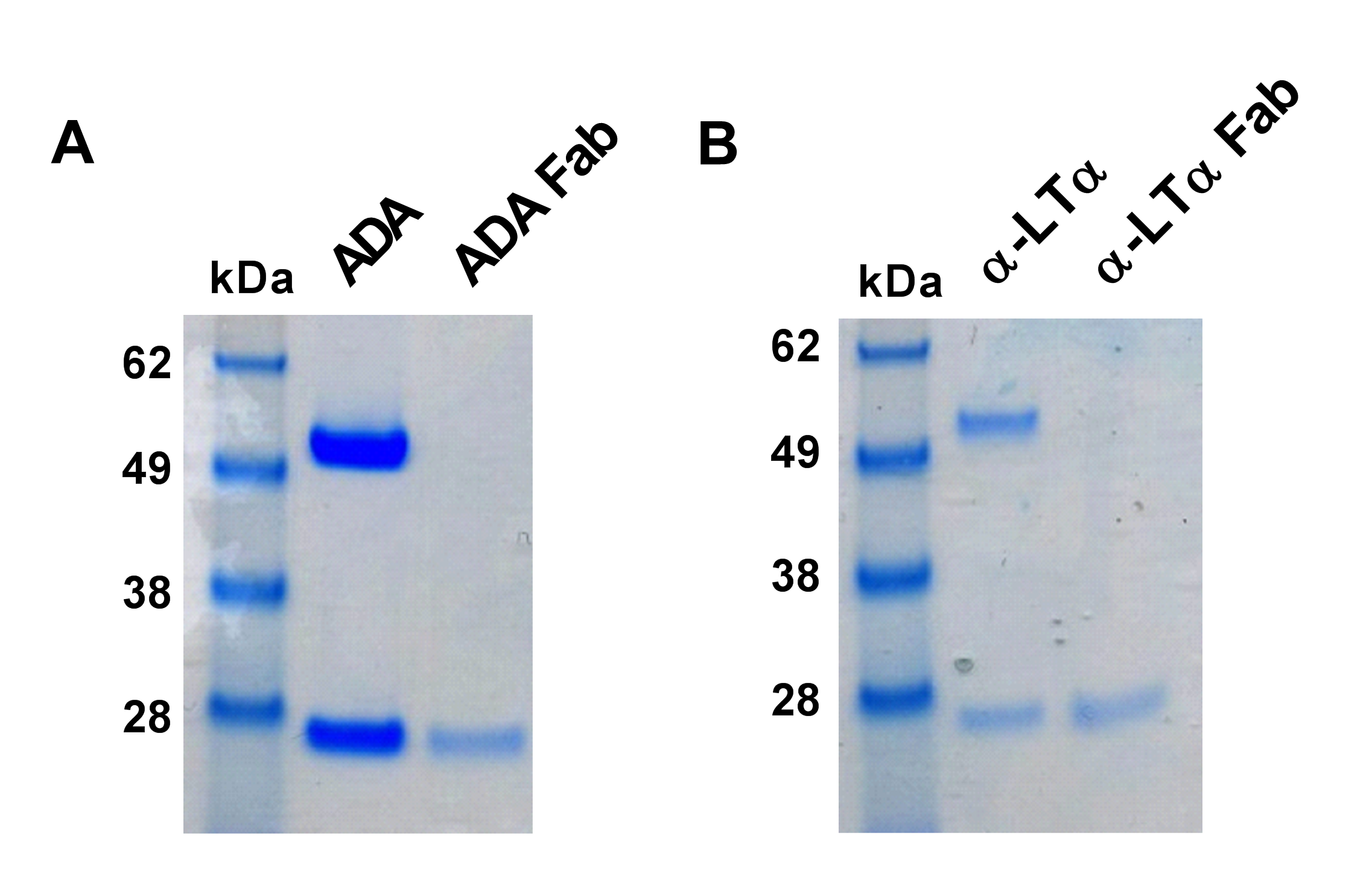

Supplement: S3 Fig — SDS-PAGE and Coomasie blue staining of adalimumab (ADA) (A) or an anti-LT-α antibody (B) and their purified Fab fragments (ADA-Fab and α-LT-α-Fab, respectively). (TIF) [file ppat.1008312.s003.tif]

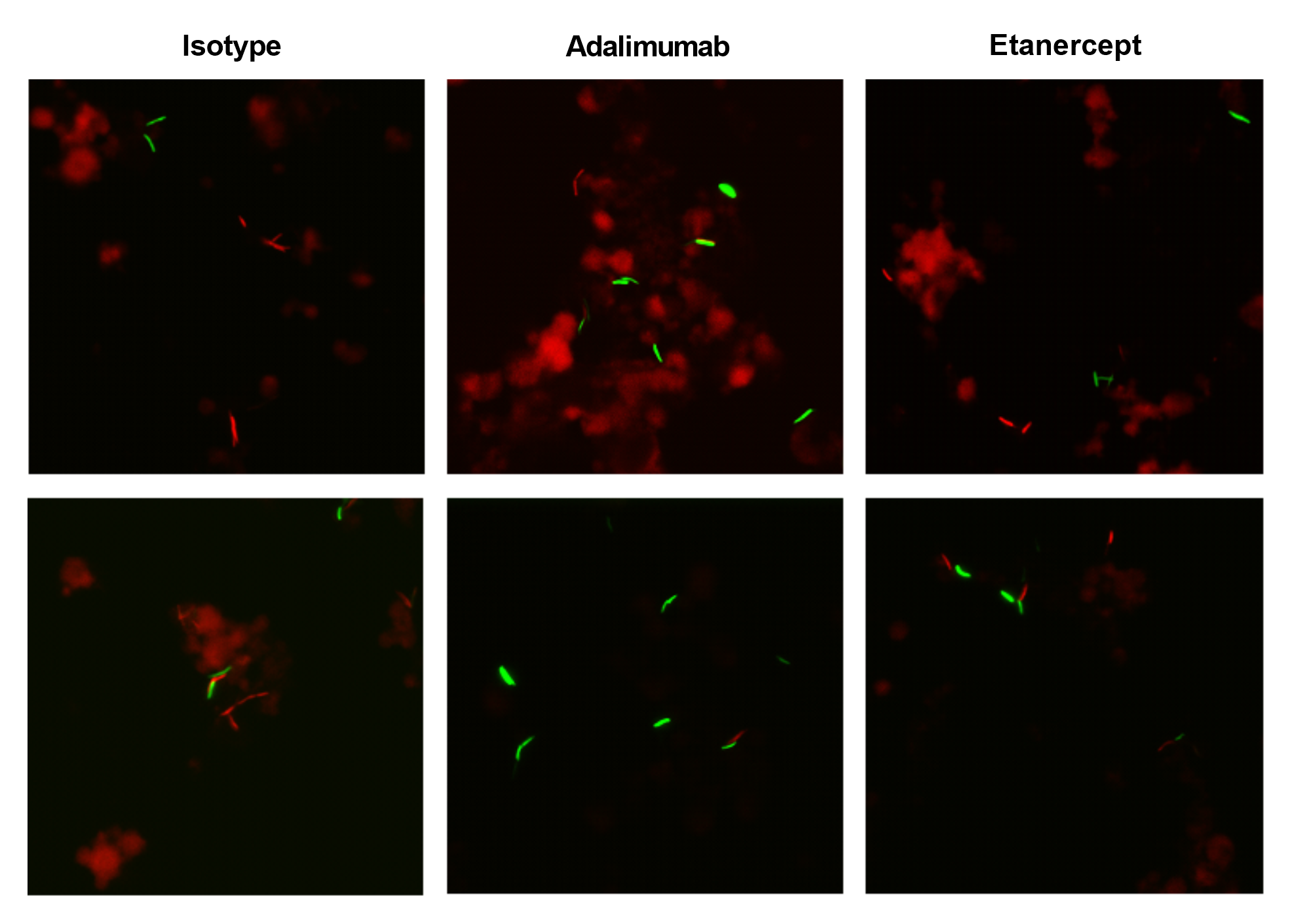

Supplement: S4 Fig — Mtb H37Rv recovered from granulomas 8 days post-infection and after 4 days of exposure to either adalimumab, etanercept or an isotype control. (TIF) [file ppat.1008312.s004.tif]
