# Supplementary material for: TNF-α antagonists differentially induce TGF-β1-dependent resuscitation of dormant-like Mycobacterium tuberculosis
Source: PLoS Pathog. 2020 Feb 18;16(2):e1008312. doi: 10.1371/journal.ppat.1008312 (PMC7048311; doi:10.1371/journal.ppat.1008312)
Supplement: S1 Table — (PDF) [file ppat.1008312.s005.pdf]

S1 Table. Sequences and references of the primers used for qPCR

Gene	Primer sequence (5'→3')	Reference
icl1 (Rv0467)	CCAGAACCAGATGAGCGCGTAT<br>GGCCCTCTTCGGTGGAAC	[1]
gltA1/prpC (Rv1131)	ATTCGTTGACCTACCGGGGATA<br>CAGCATCGAGCGGTCCAC	[2]
nuoB (Rv3146)	CGAGAAGGTGGCGGGCTATGTC<br>GAACCGCGCAATGTCAAACCTTG	[3]
ctaD (Rv3043c)	GCGTCGCGTGCATAAGCTTTT<br>GCCGTGCATGGTGAACAACTG	[3]
16S rRNA	GTGATCTGCCCTGCACTTC<br>ATCCCACACCGCTAAAGCG	[4]

1. Shi L, Sohaskey CD, Pfeiffer C, Datta P, Parks M, McFadden J, et al. Carbon flux rerouting during Mycobacterium tuberculosis growth arrest. Mol Microbiol. 2010;78(5): 1199-215. pmid:21091505
2. Banerjee SK, Kumar M, Alokam R, Sharma AK, Chatterjee A, Kumar R, et al. Targeting multiple response regulators of Mycobacterium tuberculosis augments the host immune response to infection. Sci Rep. 2016; 6:25851. pmid: 27181265
3. Shi L, Sohaskey CD, Kana BD, Dawes S, North RJ, Mizrahi V, Gennaro ML. Changes in energy metabolism of Mycobacterium tuberculosis in mouse lung and under in vitro conditions affecting aerobic respiration. Proc Natl Acad Sci U S A. 2005;102(43): 15629-34. pmid:16227431
4. Honeyborne I, McHugh TD, Phillips PP, Bannoo S, Bateson A, Carroll N, et al. Molecular bacterial load assay, a culture-free biomarker for rapid and accurate quantification of sputum Mycobacterium tuberculosis bacillary load during treatment. J Clin Microbiol. 2011;49(11): 3905-11. pmid:21900522
