# Supplementary material for: TNF-α antagonists differentially induce TGF-β1-dependent resuscitation of dormant-like Mycobacterium tuberculosis
Source: PLoS Pathog. 2020 Feb 18;16(2):e1008312. doi: 10.1371/journal.ppat.1008312 (PMC7048311; doi:10.1371/journal.ppat.1008312)
Supplement: S2 Table — Statistical analysis was performed using a generalized linear mixed-effects model; n.s., not significant; *, p<0.05, **, p<0.01; ***, p<0.001; ****, p<0.0001. (PDF) [file ppat.1008312.s006.pdf]

S2 Table. Statistical analysis of Fig 5 C-D

(A)

vs.	Iso	Iso + α-TGF-β1	ADA	ADA + α-TGF-β1	ETA	ETA + α-TGF-β1
**Iso**						
**Iso + α-TGF-β1**	n.s.					
**ADA**	****	****				
**ADA + α-TGF-β1**	n.s.	n.s.	****			
**ETA**	*	*	***	*		
**ETA + α-TGF-β1**	*	*	****	n.s. (0.053)	n.s.	

(B)

vs.	Iso	ADA	ADA + α-TGF-β1	ADA Fab	ADA Fab + α-TGF-β1	ETA
**Iso**						
**ADA**	****					
**ADA + α-TGF-β1**	n.s.	****				
**ADA Fab**	***	**	*			
**ADA Fab + α-TGF-β1**	**	**	n.s.	n.s.		
**ETA**	*	***	n.s.	n.s.	n.s.	
