# Supplementary material for: TNF-α antagonists differentially induce TGF-β1-dependent resuscitation of dormant-like Mycobacterium tuberculosis
Source: PLoS Pathog. 2020 Feb 18;16(2):e1008312. doi: 10.1371/journal.ppat.1008312 (PMC7048311; doi:10.1371/journal.ppat.1008312)
Supplement: Attachment — Submitted filename: Response to Reviewers.docx [file ppat.1008312.s008.docx]

**Reviewer #1:**

**Part I - Summary**

The author reports use of a culture system for aggregates of PBMC described as “in vitro granulomas” to investigate the differential risk of TB progression with several disease modifying antirheumatic drugs. The study addresses a clinically relevant gap in knowledge and the results for the most part parallel differences identified in clinical studies, particularly for ADA vs ETA. The work represents a novel use of the culture system for comparative studies of different biological agents and provides evidence for a TB reactivation pathway mediated by TGF-beta following crosslinking of tmTNF-alpha by ADA, as well as a previously unsuspected role for LT- alpha in maintaining latency. The author contends that the in vitro culture system provides an efficient model to assess TB risk of immune-modulating biological therapies, although the mechanism of the putative dormancy that is present in >20% of bacilli at day 1 and increases to >40% by day 8 in untreated cultures is unclear and might not reflect the mechanisms that enforce latency in vivo.

While the execution of experiments appears to have been robust, the author relies heavily on differential staining with Auramine O and Nile red to discriminate between metabolically active and “dormant” bacilli. As note in Part II, it can be argued that this approach lacks stringency. Furthermore, while the trends for changes in the proportions Auramine O and Nile red bacilli following treatment with ADA, ETA, UST and ANA were statistically significant and to a certain extent match the clinical data, the small magnitude of absolute differences between these agents in this assay raise questions about biological significance. This concern is even greater for the measure of relative rifampin resistance between ADA and ETA.

**Part II – Major Issues: Key Experiments Required for Acceptance**

Differential staining with Auramine O and Nile red is used to discriminate between biologically active vs dormant Mtb, but it is unclear whether conclusions regarding metabolism can be reliably drawn. Non-replicating persistence induced through oxygen starvation results in Mtb cells that have lipid bodies that stain with Nile red, but this can occur in a variety of conditions and might not accurately report cellular metabolic status. Similar considerations apply to Auramine O staining, which was reported to be unaffected by antimicrobial treatment (Kamariza et al. Sci Translat Med 10[430]: eaam6310). The author might consider additional tests to demonstrate dormancy, such as inability to grow on agar but retention of the capacity to grow in permissive liquid media. Another option would be Mtb gene expression profiling, as reported to Kapoor et al. (PLoS One 8[1]:e53657) who were the first to show that neutralizing TNF-alpha reduced the proportion of bacilli with features of dormancy in the in vitro granuloma model.

Author’s response: The dormancy induction and characteristics of *Mtb* physiology in this model, including gene expression profiling as well as antibiotic tolerance, had been indeed first described by Kollatukudy’s group in 2013. Originally, we did not intend to re-assess these previously published data. Nonetheless, we appreciate the reviewer’s concerns about our capacity to demonstrate that the system behaves as previously described in our laboratory setting. In current Fig 1 (panel D), we have now added gene expression data demonstrating that the accumulation of Nile red-positive bacteria upon granuloma formation correlates with an increased expression of dormancy/hypoxia related genes as well as reduced expression of genes associated with bacterial respiration previously reported in *in-vitro* experiments and in clinical specimens. In addition, we have now added assessment of the bacterial load by CFU at the end-point of granuloma treatment (current Fig 3C) showing that the reduction of Nile red-positive bacteria observed upon exposure to Adalimumab correlates with a significant absolute increase in the bacterial load compared to the isotype control-treated granulomas. More generally, we have tempered our wording using “dormant-like” terminology throughout the manuscript including the title.

Only PBMC samples containing CD4+ T cells that made IFN-gamma in response to PPD were used for these experiments, yet the author states that none of the donors displayed signs of LTBI based on the absence of response to immunodominant Mtb peptides. What is the basis for IFN-gamma production by CD4+ T cells from TB-naïve donors? Since the culture system meant to model immunologically enforced dormancy as occurs in LTBI, why not use donors with LTBI. Differences in cultures from donors with or without LTBI might be informative.

Author’s response: Investigating the differential behavior of this *in-vitro* granuloma model using PBMCs obtained from individuals with a different history of exposure to *Mtb* (resisters, latent, incipient, active or cured) would definitely be interesting, although somewhat difficult to interpret for latent TB constitutes in reality a spectrum of diseases much more complex than the sole detection of IFN-γ in response to a given peptide (10.1038/nrmicro2236). In addition, this has already been approached in a 2D system elsewhere (10.1128/mBio.02537-14).

Regarding the basis for IFN-γ production by CD4^+^ T cells from *Mtb*-naïve donors. As some of the compounds used in this study are targeting cytokines produced by T cells, namely TNF-α and IL-17, we ought to make sure that such cells would be elicited in this model. Consequently, we screened anonymous blood donors to select those displaying evidence of T-cell immune memory classically reflected by the production of IFN-γ in response to mycobacterial antigens. Although none reacted to the *Mtb*-specific peptide pool (ESAT-6, CFP-10 and TB7.7) some did react to PPD and were selected for the study. We can reasonably assume that responses to PPD can either be attributed to the cross-reactivity of antigens delivered through previous *M. bovis* BCG vaccination and/or to previous exposure to environmental mycobacteria. This aspect has been reinforced in the revised version of our manuscript (lines 150-152).

Despite case reports of TB progression following treatment with UST, the evidence that this is a potent effect in humans is weak (Cantini et al. Mediators of Inflamm 2017:8909834). The finding of high activity of UST in the Auramine O/Nile red assay despite very low levels of IL-12p70 and IL-23 in the cultures is mechanistically unexplained and raises some concern about the validity of the model, at least for testing this particular biological agent.

Author’s response: The finding of a high activity for UST in the Auramine O/Nile red assay despite very low levels of IL-12p70 and IL-23 in the cultures can be readily explained and actually mechanistically supported by Mendelian susceptibility to mycobacterial disease due to mutation in the IL12p40 gene or the beta chain of its receptor ([10.1016/j.smim.2006.07.010](https://doi.org/10.1016/j.smim.2006.07.010)). Contrary to raising concerns about the validity of the model, we think that it shows that this system is able to capture the consequence of the neutralization of a key cytokine that does not accumulate and that would be neglected if one would attempt to raise conclusions on its importance only based on its sole prevalence. For instance, IL-10 also acts at very low concentration and is hardly detected during *Mtb* infection by standard methods. Actually, this forced the group of Dr. O’Garra to develop IL-10 reporter mice to elucidate the kinetics and cellular sources of IL-10 that would hamper the control of *Mtb* infection (doi: 10.4049/jimmunol.1601340). This has now been further discussed in the revised version of the manuscript (lines 412-414).

**Part III – Minor Issues: Editorial and Data Presentation Modifications**

The seven paragraph Introduction and the Discussion could be trimmed without loss of information. The author should work to avoid redundancies between these two sections but should also consider an expanded discussion of potential weaknesses in vitro culture system as a model of granulomas forming in vivo.

Author’s response: We apologize for leaving behind these redundancies. We have reviewed these sections removing notably several repetitions around the intrinsic structural and functional differences distinguishing ADA and ETA. We hope the flow of the manuscript has now improved.

Regarding the potential weaknesses of *in-vitro* culture system as a model of granulomas forming *in vivo*, a comprehensive review was recently released in the Journal of Infectious Diseases listing potential as well as limitations and weaknesses of the various attempts to model granulomas *in vitro* including the approach used in the study presented here (10.1093/infdis/jiz020). We are now referring to these limitations in the discussion of the revised manuscript (lines 425-428).

The author might consider a supplemental figure with micrographs to allay any concerns about the accurate discrimination between Auramine O vs Nile red positive bacterial cells. If there is any ambiguity in staining, then it would be necessary for the population counts to be performed by a blinded reader.

Author’s response: As suggested, we have added a supplemental figure (S4 Fig) including micrographs of representative views of Auramine and Nile red positive bacterial cells in our experimental setting to illustrate that the data presented here were generated without ambiguity.

**Reviewer #2:**

**Part I - Summary**

The work from Arbues et al. builds on a model in which human PBMCs are assembled into granuloma-like structures, previously described in 2013 from Kalattakudy's group to examine the effect of various biologics targeting TNF. They carry out many of the same experiments conceptually as the 2013 paper, including monitoring of auramine/nile red ratio, as a measure of metabolic activity of the bacteria. They then go on to test a number of biologics that are used clinically and reproduce a difference in the effects of two drugs that depends on the mechanism of action. This is a clever use of these assays, the experiments are generally well carried out, and they seem to have uncovered some differences in mechanism and the interventions. My major concern is that almost everything relies on a single assay (auramine/nile red ratio) which the authors define somewhat arbitrarily as latency/dormancy. At minimum, there needs to be additional support for the claims around dormancy as well as consideration (cfu, bacterial numbers) of overall effects on CFU in this model.

Many of the differences shown (e.g. TGFbeta) are quite small in terms of the overall changes in ratio so, while interesting, it feels a bit dangerous to extrapolate from a single ratio-based readout in a single technical approach.

**Part II – Major Issues: Key Experiments Required for Acceptance**

As I wrote above, the extrapolation to latency/dormancy/reactivation in the time frame and ratio-based readout of the experiments seems a bit of a stretch.

1) There needs to be some investigation and validation of what the nile red/auramine populations are in terms of dormancy/latency. What is the justification for calling nile red positive bacteria truly dormant? It is also strange to me (see point below) that everything is graphed as ratios. While there may be some justification for this in terms of the complexity/scoring of the assay, it seems to me important to know what the absolute numbers of bacilli are with each treatment.

Author’s response: The dormancy induction and characteristics of *Mtb* physiology in this model, including gene expression profiling as well as antibiotic tolerance, had been described by Kollatukudy’s group in 2013. Originally, we did not intend to re-assess all previously published data. Nonetheless, we appreciate the relevance of the reviewer’s concerns as to demonstrate that the system behaves as previously described in our laboratory setting. In current Fig 1 (panel D), we have now added gene expression data demonstrating that the accumulation of Nile red-positive bacteria upon granuloma formation correlates with an increased expression of dormancy/hypoxia-related genes as well as reduced expression of genes associated with bacterial respiration previously reported in *in-vitro* experiments and in clinical specimens. More generally, we have tempered our wording using “dormant-like” terminology throughout the manuscript including the title.

2) I think there needs to be some cfu data presented along with the ratio readouts to go along with these assays. The ratio is a useful metric, but could mean quite different things depending on what happens to overall numbers of bacteria in each condition.

Author’s response: As requested, we have now added assessment of the bacterial load by CFU at the end-point of granuloma treatment (current Fig 3C) showing that the accumulation of Auramine-positive bacteria observed upon exposure to Adalimumab correlates with a significant absolute increase in the bacterial load compared to the isotype control-treated granulomas.

**Part III – Minor Issues: Editorial and Data Presentation Modifications**

I think the authors need to be more precise in defining what they have shown. There have been long discussions about what constitutes latency and dormancy and reactivation in tuberculosis, and the simplified view throughout the paper is that dormancy simply corresponds to a nile red postive population. The writing should be adjusted accordingly throughout and, as mentioned above, these bacterial populations should be probed in some other way experimentally.

Authors’ response: As answered above, we have probed in another experimental way the bacterial populations and added gene expression data (current Fig 1D), demonstrating that the accumulation of Nile red-positive bacteria upon granuloma formation correlates with an increased expression of dormancy/hypoxia-related genes as well as reduced expression of genes associated with bacterial respiration. More generally, we have tempered our wording using “dormant-like” terminology throughout the manuscript (including the title) and use “resuscitation or reactivation” and “dormancy versus latency” appropriately together with references and notably the recent review of Dr. Kaushal on this matter.

How do these data correspond with genetic manipulations of the TNF axis? Are there genetic manipulations that could be achieved in this model that would corroborate the findings from the biologics targeting the TNF pathway, particularly in regards to TGFb, which is an interesting finding but with rather mild effects in this model.

Authors’ response: Our data do not suggest that TGF-β only plays a mild effect in the resuscitation of *Mtb* mediated by Adalimumab for it accounts for 50% of its activity. The suggested genetic manipulation in regards to TGF-β in particular may consist of performing an RNA interference experiment to distinguish between *de novo* production of TGF-β and an increased conversion of the latent form present in human serum. However, transfection of *in-vitro* granuloma embedded in a collagen matrix seems technologically challenging if possible at all. Nonetheless, we are now discussing this possibility (lines 361-364).

**Reviewer #3:**

**Part I - Summary**

This manuscript by Arbues et al., explores the mechanism of TNF antagonist-mediated Mycobacterium tuberculosis (Mtb) reactivation. Using an in vitro granuloma model, they test a panel of immunomodulators currently in clinical use to determine their effects on Mtb reactivation in this model system. They show that two TNF-neutralizing biologics showed differential effects in their system. Adalimumab promoted Mtb Auramine-O staining, which is interpreted as a readout for active Mtb metabolism/replication, whereas etanercept induced more Mtb Nile red staining, which is taken as indicative of dormancy. These observations, as interpreted, parallel the clinical scenario, in which Adalimumab treatment results in a higher risk of Mtb reactivaton than Etanercept treatment. They went on to show that these effects were not mediated by TNF neutralization, but rather, were due to reverse signaling through membrane TNF, by Adalimumab (which is bivalent), but not by Etanercept (which is monovalent) and that this signaling induced TGFb, which in turn was responsible for the reactivation. This is a novel idea, which if true, would be an important finding for the field. However, the data presented, relying exclusively on these staining approaches without showing validation that these staining patterns represent increased replication or bacterial loads, is not sufficient to convincingly support their findings. More experimental evidence is needed to bolster their conclusions.

**Part II – Major Issues: Key Experiments Required for Acceptance**

1) Prior work by other groups working with the in vitro granuloma model have used immunofluorescent antibody staining to show that the immune aggregates formed in vitro bear some features of in vivo granulomas. However, in this paper the authors show only a bright field aggregate of cells. The authors should show by immunofluorescence that these aggregrates, in their own hands, show organizational features that resemble granulomas.

Authors’ response: Organizational features of granuloma consist of a central zone composed of macrophages and giant cells surrounded by leukocytes. Following reviewer’s suggestion and consistently with the previous report from Kapoor *et al*., we used live cell-tracker dyes to show that these structures consist of monocyte-derived macrophages (orange) surrounded by T cells (green) as well as additional unlabeled mononuclear-cell subsets recalling the organizational features of *in-vivo* granulomas. These experiments are shown as an additional panel (panel B) within current Fig 1.

2) The manuscript’s conclusions depend entirely on the postulate that Auramine-O staining indicates metablically active/replicating bacteria whereas Nile red staining represents dormant bacteria, however, no experimental evidence is provided to validate these assumptions. This is essential. In addition, bacterial load determinations should also be shown. If Mtb has reactivated in Adalimumab-treated granulomas, the CFUs should be higher.

Authors’ response: As requested, we have now added assessment of the bacterial load by CFU at the end-point of granuloma treatment (current Fig 3C) showing that the accumulation of Auramine-positive bacteria observed upon exposure to Adalimumab correlates with a significant absolute increase in the bacterial load compared to the isotype control-treated granulomas.

3) Fig. 5 depicts the effects of lymphotoxin neutralization on auromine-O and Nile red staining. These experiments seem only loosely related, at best, to the studies of the TNF antagonists and their differential impact of TGFb production via membrane TNF signaling. It is unclear what value these data add to this manuscript. The manuscript would be strengthened by bolstering the experiments regarding the TNF antagonists and their differential impact on TGFb and reactivation (as discussed above) and by eliminating these LT experiments (which are unrelated, and less developed). As is, seems like an odd way to end the paper.

Authors’ response: We regret that the importance of this experiment did not transpire. In this study, we aim to distinguish the mechanism of actions of so-called TNF-α antagonists that are clinically favoring LTBI reactivation. We show that while adalimumab triggers it through TGF-β production, etanercept does it through the neutralization of TNF-α as well as TNF-β independently of TGF-β. This piece of data appears in line with our objectives and has been acknowledged as an important finding by reviewer 4. Taken together, we hope the reviewer will accept that this work can remain in the manuscript.

4) In the Introduction (lines 88-96) discuss previous experimental studies that attempt to define the mechanism that could be responsible for the differential risk of reactivation observed between anti-TNF antibodies and the receptor fusion protein, however, the authors overlook an intriguing study be Steffen Stenger’s group which may be relevant to their own findings (PMID: 19381021). In this study, the author’s observed lower numbers of effector T cells that expressed effector molecules (perforin and granulysin) in individuals treated with anti-TNF antibodies compared to those treated with receptor fusion proteins. They showed that antibodies could mediated complement-mediated lysis of effector T cells that expressed surface TNF, whereas the receptor fusion proteins could not, and suggested that this could explain the differential effect on reactivation (if effector T cells were critical for holding in check). However, the reduction in effector T cells could alternatively be due to intrinsic TGFb produced by reverse membrane TNF signaling. This seems worthy to include both in the Introduction and the Discussion if the findings can be validated and shown convincingly.

Author’s response: We apologize for having overlooked this piece of work. The complement-mediated lysis of effector T cells that expressed surface TNF-α is unlikely playing a role in our model for our protocol uses de-complemented human serum. Besides, this report lead the authors to support “a rationale for determining whether TEMRA cells are preserved during etanercept treatment”. Yet, no report refuted that TEMRA cells could be naturally enriched in RA and AS patients independent of having received antibodies or receptor fusion proteins directed against TNF-α. Nonetheless, a reduction in effector T cells due to intrinsic TGF-β produced by reverse signaling via tmTNF-α may add-up to TGF-β regulation of macrophage functions to contribute to the preferential resuscitation of *Mtb* in patients treated with ADA compared to ETA and we are now discussing this aspect in the discussion of the revised manuscript (lines 367-373).

**Part III – Minor Issues: Editorial and Data Presentation Modifications**

1) In the first paragraph of the introduction, the authors go part way in their discussion of their discussion of “LTBI”. However, I think it is important that the field discuss the definition of LTBI more directly. I suggest something like, “its estimated that one quarter of the world has immunological evidence of past infection with Mtb. Although this population is frequently said to have LTBI, the % that truly harbor Mtb is not clear. These individuals likely have a range of outcomes of infection, including cure or eradication, etc. etc.

Author’s response: Thank you for this relevant suggestion. We have amended this part of the introduction accordingly.

2) The sentence in lines 51-53 is likely inaccurate (5-10% progression from latent to active when immune system is weakened). Because most of these cases occur in regions where Mtb transmission is frequent, and several studies in these setting suggest that most of these are not reactivation events, but represent recent infections.

Author’s response: Thank you once more. We have also amended this part of the introduction accordingly.

3) In discussing Fig 2A, the authors conclude that TNF production is bi-modal with one peak at day 1 post-infection and the other at day 8. However, no statistics are provided and the differences do not appear significant. This analysis needs to be bolstered with statistics, or the conclusions should be softened.

Author’s response: We have now added the requested appropriate statistics in the revised version of our manuscript showing significant p-values consistent with a bi-modal production of TNF-α. In addition, we have now modified the figure pairing data points for each donor with a line (see current Fig 3A).

4) The difference in Rif-tolerant Mtb between ADA and ETA may be statistically significant (Fig. 2C), but are very, very modest, and of questionable biologic importance. These conclusions should be softened.

Author’s response: As emphasized, the difference in Rif-tolerant *Mtb* between ADA and ETA (current Fig 3D) may seem modest but actually very strongly supported by statistics. Nonetheless, we have now softened our conclusions in the revised version of our manuscript (line 233).

5) Figure 2D is very difficult to interpret because the various lines on the graph are not labeled or defined in the figure legend. Same comment for Figure 4B.

Author’s response: We apologize for the omission of this information; we have now updated the labels and definitions on the legend text for these figures (current Fig 3E and Fig 5B) in the revised version of our manuscript.

6) The difference between Iso and ADA in Fig. 4A does not appear statistically significant. The author’s report that it is significant using Friedman’s test? Is this the most appropriate test to use here? Is this a paired analysis? If so, should the data points from a single patient be connected by lines or color-coded?

Author’s response: We thank the reviewer for pointing out that Friedman’s test may not be the most appropriate test to be used in the context of former Fig 4A (current Fig 5A). The statistical analysis has been repeated using a paired t test (after testing the residuals normality using the Shapiro-Wilk test) obtaining comparable results. In addition and as suggested, we have now modified the figure pairing data points for each donor by a line.

7) In line 312, “we present a tissue of evidence...” Is this a typo?

Author’s response: The sentence has been modified for clarity purposes (line 343).

**Reviewer #4:**

**Part I - Summary**

This study uses an in vitro model of human T cell activation by very low dose M. tuberculosis (M.tb) infection of PBMC within an extracellular matrix of collagen and fibronectin to analyse the effect of different anti-cytokine “biologics” on “reactivation” of M.tb as measured by the ratio of acid-fast and lipid containing mycobacteria. They infer this is equivalent of chronic granulomas in humans with latent TB infection (LTBI), but this is an overstatement of the significance of the model. Nevertheless, they make interesting and novel observations on the differential capacity of different anti-cytokine therapies to increase the recovery of acid-fast M.tb that they infer are metabolically active. They propose that reverse signalling through memTNF receptors on macrophages by bivalent anti-TNF mAb (ADA) stimulates TGF-b production that contributes to the increased potential of ADA to cause “reactivation” compared to soluble TNFR2 inhibitor (ETA). This is supported by effect of anti-TGF-b antibodies, but there are some inconsistencies in the data that should be addressed. They also highlight the effect of another TNFSF member, LT-a, in preventing reactivation.

1. Model:

The cellular aggregates of T cells and macrophages are followed for 8 days only and do not have all the hallmarks of chronic granulomas in humans with LTBI, eg epithelioid macrophages, giant cells and fibrosis from mesenchymal cells. Therefore, although a useful model, it is not equivalent to chronic granulomas, and this should be acknowledged and discussed. The “reactivation” is based on ratio of acid-fast mycobacteria to those containing lipid bodies over 8 days. Have they or others demonstrated differences in activation of dormancy genes in this model? Does the addition of anti-TNF mAb lead to increased numbers of bacteria cultured from the cellular aggregates? Results from supporting mycobacterial cultures would strengthen the findings from the model.

Authors’ response: We agree that one should highlight the limitation of this granuloma model that indeed lacks non-hematopoietic derived cells, vascularization and plasticity; i.e. continuous influx of freshly recruited cells. This has now been acknowledged and discussed in the revised version of the manuscript (line 425-428).

Concerning the question of the activation of dormancy genes in this model, this had been described by Kollatukudy’s group in 2013. Originally, we did not intend to re-assess these previously published data. Nonetheless, we appreciate the relevance of the reviewer’s concerns as to demonstrate that the system behaves as previously described in our laboratory setting. In current Fig 1D, we have now added gene expression data demonstrating that the accumulation of Nile red-positive bacteria upon granuloma formation correlates with an increased expression of dormancy/hypoxia-related genes as well as reduced expression of genes associated with bacterial respiration as described *in vitro* and in clinical specimens.

With regards to the bacterial load aspect, we have now added CFU assessment at the end-point of granuloma treatment (current Fig 3C) showing that the accumulation of Auramine-positive bacteria observed upon exposure to Adalimumab correlates with a significant absolute increase in the bacterial load compared to the isotype control-treated granuloma.

2. Selection of subjects:

These appeared to be from blood donors selected because of IFNg-secreting T cell response to PPD, but M.tb-specific T cell antigens. Does this mean they were BCG-vaccinated and not M.tb infected? Why were these referred to as ”pre-immune” donors (L313) when they were PPD-positive? Have they or others compared responses in well characterised subjects with definite LTBI or active TB? What is the response in this model from uninfected controls, ie PPD-negative subjects.

Authors’ response: Investigating the differential behavior of *in vitro* granuloma models using PBMCs obtained from individuals with a different history of exposure to *Mtb* (resisters, latent, incipient, active or cured) may certainly deliver interesting data. However, it would be somewhat difficult to interpret such results for latent TB constitutes in reality a spectrum of diseases much more complex than the sole detection of IFN-γ in response to a peptide pool (10.1038/nrmicro2236). In addition, this has somewhat been approached in a 2D system elsewhere (10.1128/mBio.02537-14).

In this study, we aimed to investigate how the use of various biotherapeutics could interfere with the immune response ongoing during in vitro granuloma formation and maintenance and, how this interference would affect the lipid metabolism of *Mtb* which accumulation was shown to correlate with a dormant-like phenotype. Since some of the compounds used in this study are targeting cytokines notably produced by T cells, namely TNF-α and IL-17, we ought to make sure that such cells would be elicited in this model. Consequently, we screened anonymous blood donors to select those displaying evidence of T-cell immune memory classically reflected by the production of IFN-γ in response to mycobacterial antigens. Although none reacted to the *Mtb*-specific peptide pool (ESAT-6, CFP-10 and TB7.7) some did react to PPD and were selected for the study. We assume that responses to PPD can either be attributed to the cross-reactivity of antigens delivered through previous *M. bovis* BCG vaccination and/or to previous exposure to environmental mycobacteria. This aspect has been reinforced in the revised version of our manuscript (lines 150-152).

3. Results

- The effect of ADA in system was reproducible in different experiments, but ETA had stronger effect on “reactivation” in some experiments eg Fig 1C than in others, eg Fig 2, 4 and 5. Although anti-IL-17 had no effect in vitro, it may have an effect on granulomas within the lungs, and this difference should be recognised.

Authors’ response: Indeed, the baseline ratio of Auramine/Nile red positive bacteria induced upon granuloma formation in the absence of drugs (Isotype) fluctuates between independent experiments and has repercussions on the ratio observed for the tested drugs. While the resuscitation rate in the presence of ADA tend to reach a plateau across different experiments, the intermediate bioactivity of ETA appears more fluctuant across independent experiments, yet it remained always statistically significant with the isotype control- or the ADA-treated samples. This has been added in the revised version of our manuscript (lines 221-225).

Adding to our previous comment on the lack of neutrophils in this *in-vitro* model, we are now also acknowledging that although anti-IL-17 had no effect *in vitro*, we cannot rule out it may have an effect on granulomas *in vivo* (line 428-430).

- The number of donors in each figures should be clarified, eg Fig 2C, are biological replicates the same as donors or multiple samples from the same donor; and how many technical replicates were tested?

Authors’ response: The number of independent donors (biological replicates) used to generate each figure is now clearly indicated in the corresponding legend. In most cases, a single technical replicate per donor and condition was tested to generate each figure. However, there were some exceptions, e.g. for CFU assessment on current Fig 3C, two wells (technical replicates) were tested per donor and condition and were then plated in triplicates. This aspect has been included now in the Quantification and Statistical analysis paragraph of the Materials and Methods section of our manuscript.

- Fig 2 D: the ADA and ETA samples are not labelled. Were these the results from only 2 independent replicates and if so the SEM cannot be calculated?

Authors’ response: We apologize for the omission of this information and we have now updated the labels and definitions on the legend text for former Fig 2D (current Fig 3E). The results plotted on this figure were indeed generated from 2 independent donors (biological replicates). However, according to GraphPad’s article concerning statistics with n=2 (knowledgebase – article #591) it is valid to calculate SEM of out two values.

- The levels of TGF-β measured (from only 4 donors) were small and there was no difference between ADA and ETA samples that does not support their hypothesis (Fig 4A), although exogenous TGF-b at higher concentrations did have an effect.

Authors’ response: The levels of TGF-β that we measured are indeed very small and actually close to the limit of detection. Even though, there is no statistical difference between ADA and ETA, there is also no statistical difference between Iso and ETA. This brought us to think in the first place that ADA was inducing more TGF-β than ETA which would led to the more pronounced bioactivity. However, the blocking experiments performed (n=8 in this case, current Fig 5C-D) clearly revealed that TGF-β only plays a role in the context of ADA treatment. We have update our sentence (from line 265 onwards) as follows: “We consequently aimed to address if such a concentration of TGF-β1 could interfere with granuloma-induced *Mtb* dormancy and lead to mycobacterial resuscitation. […] As depicted in Fig 5B, the presence of exogenous TGF-β1 at 8 pg/ml was already sufficient to significantly impact *Mtb* dormancy”.

- The effect of anti-LTa in their model (Fig 5) does support a role for T-cell derived LTa on control of M.tb infection. But they should explain the statement about the “neutralization of one TNFR2 ligands somewhat interferes with the sensing of the other”(L297).

Authors’ response: We can only speculate here but as we could not detect additive activity of blocking both TNF-α and LT-α, it may reflect that the receptor somewhat still interact with one or the other cytokine while bound to the Fab fragment. As such, this may compete with the binding of the other cytokine or indirectly prevent its signaling after internalization of the receptor. We are now discussing this possibility in the revised version of the manuscript (lines 383-386).

4. Statistical analysis

Different statistical methods were used in analysis of different results. Why was a generalized liner mixed-effects model used rather than a simpler non-parametric comparison between groups in some and not other experiments?

Authors’ response: The generalized linear mixed-effects model was solely used when appropriate i.e. all experiments comparing the representation of Auramine versus Nile red positive bacteria across various treatments and donors. This approach was advised and designed by our internal statistics expert (Dr. Schindler Christian, cited in the acknowledgment) to account for the number of individual bacteria that were counted by microscopy (>200 per sample; and that percentages would minimize) and to account for the number of biological replicates performed (number of donors) as well as the number of treatments to include a multiple testing correction step.

**Part II – Major Issues: Key Experiments Required for Acceptance**

1. Does the addition of anti-TNF mAb lead to increased numbers of bacteria cultured from the cellular aggregates? Results from supporting mycobacterial cultures would strengthen the findings from the model.

Authors’ response: As discussed above, we have now included CFU assessment at the end-point of granuloma treatment (current Fig 3C) showing that the accumulation of Auramine-positive bacteria observed upon exposure to Adalimumab correlates with a significant absolute increase in the bacterial load compared to the isotype control-treated granulomas.

2. The levels of TGF-β in ADA and ETA inhibited samples were measured in only 4 donors and were small. There was no difference between ADA and ETA samples that does not support their hypothesis (Fig 4A). This should be repeated from more samples.

Authors’ response: As detailed above, the levels of TGF-β that we measured are indeed very small and actually close to the limit of detection. Even though, there is no statistical difference between ADA and ETA, there is also no statistical difference between Iso and ETA. This brought us to think in the first place that ADA was inducing more TGF-β than ETA which would led to the more pronounced bioactivity. However, the blocking experiment performed (n=8 in this case, current Fig 5C-D) clearly revealed that TGF-β only plays a role in the context of ADA treatment. We have update our sentence (from line 265 onwards) as follows: “We consequently aimed to address if such a concentration of TGF-β1 could interfere with granuloma-induced *Mtb* dormancy and lead to mycobacterial resuscitation. […] As depicted in Fig 5B, the presence of exogenous TGF-β1 at 8 pg/ml was already sufficient to significantly impact *Mtb* dormancy”.

**Part III – Minor Issues: Editorial and Data Presentation Modifications**

(No response)
